# Dichotomy in the definition of prescriptive information suggests both prescribed data and prescribed algorithms: biosemiotics applications in genomic systems

**DOI:** 10.1186/1742-4682-9-8

**Published:** 2012-03-14

**Authors:** David J D'Onofrio, David L Abel, Donald E Johnson

**Affiliations:** 1 Control Systems Modeling and Simulation, General Dynamics, Sterling Heights MI, USA and College of Arts and Science, Math Department, University of Phoenix, Detroit MI, USA; 2 Director, The Gene Emergence Project, The Origin of Life Science Foundation, Inc., 113 Hedgewood Dr., Greenbelt, MD 20770-1610 USA; 3 Retired Scientist and Professor (APU, U-MD, U-MN & U-WI), 5002 Holly Tree Rd, Wilmington, NC 28409

**Keywords:** Prescriptive Information (PI), Functional Information, algorithm, processing, language, ribosome, biocybernetics, biosemiosis, semantic information, control, regulation, automata, Frame Shift Mutation

## Abstract

The fields of molecular biology and computer science have cooperated over recent years to create a synergy between the cybernetic and biosemiotic relationship found in cellular genomics to that of information and language found in computational systems. Biological information frequently manifests its "meaning" through instruction or actual production of formal bio-function. Such information is called Prescriptive Information (PI). PI programs organize and execute a prescribed set of choices. Closer examination of this term in cellular systems has led to a dichotomy in its definition suggesting both prescribed data and prescribed algorithms are constituents of PI. This paper looks at this dichotomy as expressed in both the genetic code and in the central dogma of protein synthesis. An example of a genetic algorithm is modeled after the ribosome, and an examination of the protein synthesis process is used to differentiate PI data from PI algorithms.

## Background

Bioinformatics has opened up the field of molecular biology through the use of computer science and statistics. Data mining of genetic information includes discovering relationships between individual DNA sequences and variability in disease [1]. More importantly, the application of computer science will contribute to identifying intricate complex data and algorithmic structures that are part of the biological processes that manage and maintain metabolic functions of the cell.

Biological organisms are considered to be controlled and regulated by Functional Information (FI) [2-8]. FI comes closer to expressing the intuitive and semantic sense of the word "information" than mere Shannon combinatorial uncertainty or reduced uncertainty (poorly termed "mutual entropy"). The innumerable attempts that have been made to reduce the functional information of genomics and molecular biology to nothing more than physical combinatorics and/or thermodynamics will fail for reasons best summarized in the peer-reviewed anthology entitled *The First Gene: The Birth of Programming, Messaging and Formal Control *[9].

"Functional Information (FI)" has now been formalized into two subsets: Descriptive Information (DI) [7] and Prescriptive Information (PI) [7,10,11]. This formalization of definitions precludes the prevailing confusion of informational terms in the literature. The more specific and accurate term "Prescriptive Information (PI)" has been championed by Abel [12-16] to define the sources and nature of programming controls, regulation and algorithmic processing. Such prescriptions are ubiquitously instantiated into all known living cells [13]. PI either instructs or produces formal function [12] in such a way as to organize and institute a prescribed set of logic-gate programming choices. Without such steering of physicochemical interactions by "Choice-Contingent Causation and Control" (CCCC) [17-19], metabolic pathways and cycles would be impossible to integrate into a cooperative and holistic metabolism. The Organization (O) Principle [19] states that nontrivial formal organization can only be produced by CCCC.

Maynard Smith [20] argued that bioinformation is both specific and intentional. Maynard Smith also pointed out in this same paper the irreversibility of information transfer. Information moves only from signal to response, not in the reverse direction. He argued that genetic information implies the possibility of misinterpretation or error. Maynard Smith also considered genetic information to be undetermined by cause-and-effect necessity. But he considered genetic information to be gratuitous (not called for by the circumstances: unwarranted) [20].

Jablonka [21] argues that life is dependent upon semantic information, and that Shannon "information" is insufficient to explain life. She emphasizes, as does Adami [22], the importance of "aboutness." Aboutness relates to meaning which in biology relates to biofunction.

Jablonka [21] also argues that semantic information can only exist with living or designed systems. "Only a living system can make a source into an informational input." On page 588 Jablonka emphasizes the *function *of bioinformation. Thus the joint authors of this paper are not alone in our emphasis on the formal nature of life's many control mechanisms.

A closer examination of Prescriptive Information (PI) has led to a dichotomy in its definition to differentiate between 1) what are prescribed data, and 2) what are prescribed algorithms. As the concepts of computer science are applied to the cell, it is necessary to deconstruct information structures to identify and differentiate data from algorithms. The DNA polynucleotide molecule consists of a linear sequence of nucleotides, each representing a biological placeholder of adenine (A), cytosine (C), thymine (T) and guanine (G). This quaternary system is analogous to the base two binary scheme native to computational systems. As such, the polynucleotide sequence represents the lowest level of coded information expressed as a form of machine code. Since machine code (and/or micro code) is the lowest form of compiled computer programs, it represents the most primitive level of programming language. Typical machine code consists of single instructions which are interpreted by the microprocessor in a linear sequential program flow. In this form it is not apparent as to how to identify algorithms and data structures easily seen in higher level programming languages such as BASIC, LISP, FORTRAN and C. This is because binary machine code is a comma-less string of ones and zeros which mimics the comma-less sequence of quaternary placeholders that constitute the genome. The absence of higher level structure and visibility of interpretive language found in higher level programming formats makes the read-ability and identification of algorithmic and data structures difficult to the reader. Examination of the genomic literature offers some clues in de-ciphering this biological machine code in terms of differentiating data from algorithms. Allowing for the breakdown of PI into its constituent components may permit the identification of models that better explain the structure and process of information systems within the cell. An understanding of algorithms will be defined from a computer science perspective due to the discrete nature of cellular systems and operations. This is in part due to the cell's primary centralized genetic information source defined as DNA, consisting of both instructions and data, represented as a quadruple discrete code. Each nucleotide token can be defined as either a formal state of physicality or of abstract space.

Each Shannon bit of uncertainty represents a binary choice opportunity, not a specific choice. Shannon's famous H equation (equation 1) clearly shows that a "bit" is nothing more than the log of a probability distribution.

(1) $$H=\overset{M}{\underset{i=1}{\sum }}p_{i}\left(-\log_{2}p_{i}\right)$$

There are no specific choices to be found anywhere within this mathematical definition of a "bit." Shannon worked only on general communication engineering problems. He deliberately made no attempt to quantify intuitive/semantic information by measuring specific functional choices with fixed units. That would be impossible.

In computer science, bits are used to measure the number of binary choice *placeholders *in a *potential *digital prescriptive informational (programming) string. Even after a program is written, "bits" refer only to the total number of binary choices the program contains. Under no circumstances do "bits" identify a particular binary choice.

When we move from negative bits of uncertainty to a positive specific enumeration of particular functional choices, that is when Functional Information (FI), and its two subsets, Descriptive Information (DI) and Prescriptive Information (PI) are introduced [19].

Since each potential nucleotide selection represents one of four possible states that could be selected, two bits defined as a Dual bit (Dbit) of uncertainty exist *just prior *to each nucleotide selection at each locus in the growing biopolymeric string of potential "choices."

When a functional choice of a nucleotide is actually made, however, the polymerization of each prescriptive nucleotide into a programmed "messenger molecule" instantiates a quaternary programming choice into that syntax [12,13,23]. Each such nucleotide choice in a highly conserved gene syntax, for example, can be measured as two biological Functional bits ("Fits") of Functional Sequence Complexity (FSC) [24-26]. Two "Fits" of FSC have been formally prescribed and instantiated into that gene or edited, mature mRNA that contributes to a specified metabolic function [25,26].

The measure of FSC using "Fits" is not identical to the measure of Prescriptive Information (PI) in a program or message. The calculation of Fits, when working with proteins, for example, employs the total number of protein family members out of sequence space that display any degree of that family's biofunction. Fits do not address the degree of functionality (e.g., the catalytic constant) of any one protein in that family. But fits come the closest to measuring the *functional *uncertainty of a linear digital functional string out of sequence space of any measurement in the literature [25].

Since each nucleotide placeholder can accept one of four possible states, it comprises a logical base 4 system. (To be fully correct, we would have to include cytosine methylation as the source of an additional configurable switch-setting option, and other non-biological bases, including non-right-handed sugar nucleotides, in the alphabet of possible tokens that could theoretically polymerize onto a prebiotic string. But for simplicity at the moment, we will just think in terms of the four main biological nucleotides.) This four-state quaternary placeholder is directly analogous to the two-state binary placeholder in artificial computer systems defined as a bit. To distinguish the quaternary biological placeholder from the binary placeholder, we define the four-state biological placeholder as a Dbit (Dual bit) placeholder. The term Dbit is used to better define the differences between the biological unit from the computer unit. This kind of unitization is also seen in other fields such as quantum computing known as the Qubit. The Qubit is defined to distinguish the quantum bit from the classical computing bit.

Bit operations, whether logical, mathematical or informational, are well understood in the field of computer science, offering a rich knowledge base from which to analyze such systems. Since cell operations are dependent on Dbit recognition and consecutive step by step operations such as DNA copying processes (no new information is generated in DNA copying), mRNA editing, digital computation, protein synthesis and many more processes, these functions provide the justification to define algorithms and data from a computer science perspective. Therefore, we will define an algorithm as a set of rules and/or a step-wise procedure that precisely defines a finite sequence of operations [27]. We will discuss this in more detail in the algorithm section.

In order to differentiate between data and algorithms as it pertains to the DNA/RNA world, it is pertinent to examine languages [14], which may aid in the identification of linguistic structures as it applies to algorithms and data. This claim is supported as it relates to the computer science field of Automata Theory. Automata Theory, concerns itself with the mathematical modeling of computing functions [28] and identification of abstract languages or rules [29]. It has also been used recently in biological and biomedical systems such as autonomous DNA models, DNA sequence reconstruction and cellular level interactions [30-32]. Computing machines are modeled as mathematical abstractions, which in many ways are equivalent to real computers and programming languages [28]. These computing machines are called automata. Automata theory is also related to formal language theory.

Automata can recognize a class of formal languages given any automata or machine M that operates on symbolic characters from a given alphabet to produce language "L". This gives us a formal way to evaluate and understand machine-like operations. Automata Theory sets the precedence for applying formal language theory to modeling computing machine systems. Such computational systems are dependent upon some type of operating language, and as such, may be applicable in modeling similar biological systems. For example, automata theory has been used to model the DNA as a one dimensional cellular automaton with four states defined by its four bases [31]. This machine was evaluated to determine rules that could influence its history. We argue that the linguistic analogy for machines is not purely heuristic [14], but is necessary for physical machinery to perform computational tasks. An interesting question becomes, "Does the cell solve biological problems by equivalent methods and principles as electronic computers solve problems?"

Examination of the syntax, semantics and semiotic mechanics of linguistics has served as an abstract template when searching for similar structure in the DNA/RNA world. The field of DNA linguistics has focused on computational linguistics and molecular biology. Such efforts have contributed to developing a logic grammar formalism that has been used to perform language processing and recognition of DNA sequences such as E. coli promoters [33]. We posit that linguistic structure coupled with algorithm methodologies helps us to understand the difference between data and algorithms in the DNA/RNA world.

In order to have information transfer between two abstract spaces, there must exist a form of language that is common to each. Using concepts from automata theory as the basis of formal language, we define the following terms:

1) Symbol--an abstract placeholder with arbitrary meaning. ("Physical symbol vehicles" such as nucleotides, are called tokens).

2) Alphabet--a finite set of symbols in set Ω_dna_. (Ex. DNA nucleotides A, C, T and G)

3) Word (w)--A finite string of symbols from a given alphabet in set ∑_dna _that has semantic meaning (effects or affects bio-function).

4) Language (L)--A string of words from a given alphabet. w ∈ ∑_dna _

Language provides a protocol that has contingency and use of grammar. By grammar we mean a set of rules governing use of symbols in an effort to render symbol strings meaningful. In language, alphanumeric characters are chosen by a set of arbitrary rules such as the letter u following the letter q used in English words [23]. The language used in computing machines has been shown by Chomsky [34,35] to extend the idea of complexity hierarchy to formalized language hierarchy found in automata theory. This concept has led to the development of a formal grammar defined for computing purposes. Using grammar automata with just a few symbols and rules can produce a variety of complex languages. The transfer of information from the genome to the ribosome can be modeled using language embedded in the structure and organization of DNA/RNA and amino acids. For example, the grammatical structure of codons can be represented by the set of production rules as illustrated below:

1) S → TAA | TGA | TAG (= stop codon)

2) MMM → XXX where XXX are three arbitrary selections of the genetic DNA alphabet consisting of the letters A, C, G and T

3) S → MMMS where S is a string function that follows the rule S = the current value of MMM followed by the previous string content for S.

We execute the above rules in the following order:

Rule 1, Rule 2, Rule3, Rule2, Rule3

Rule 1 sets S equal to the stop codon string, e.g. TAA. Applying rule 2 sets MMM as any arbitrary three nucleotide selection of the genetic alphabet such as ACT or TGA, etc where X is a placeholder for an arbitrary nucleotide. Next we apply rule 3 which forms string S as S= MMMS = XXXTAA. Next we apply rule 2 again which creates another arbitrary set of codon of A's, C's T's and G's such that MMM = (XXX)_1_. Applying rule 3 again forms the string

$$\begin{array}{c} \text{S}={\left(\text{XXX}\right)}_{\text{1}}\text{XXXTAA}.\text{ Repeating rules 2}\&\text{3 produce the string}\\ \text{S}={\left(\text{XXX}\right)}_{\text{2}}{\left(\text{XXX}\right)}_{\text{1}}\text{XXXTAA} \end{array}$$

In general this grammatical rule produces a gene of arbitrary length n as

$${\left(\text{XXX}\right)}_{\text{n}}{\left(\text{XXX}\right)}_{\text{n - 1}}\cdot \cdot \cdot {\left(\text{XXX}\right)}_{\text{2}}{\left(\text{XXX}\right)}_{\text{1}}\text{XXXTAA}$$

This produces a language of genes (L) relative to the genome language L_G_. which can be represented as

(2) $$\text{L}=\left\{\left\{{\left(\text{XXX}\right)}_{\text{n}}\cdot \cdot \cdot \left(\text{stop}\;\text{codon}\right)\right\}\cdot \cdot \cdot \right\}$$

Each codon may be representative of either exons or introns. The information in equation 2 and the production rules now describe at a minimum, a subset language of genome (L_G_) expressing the coding sequence of genes.

This set of rules is by no means complete with regards to describing all of the biologic function within the genome. The authors freely acknowledge the naiveté of this model with respect to the innumerable additional dimensions of PI and layers of supplemental processing that have recently come to light in molecular biology. Nevertheless, it is necessary to begin the cybernetic comparison with linear digital prescription and the other linguistic-like parallels. For example, there would be additional rules and grammar that define the necessary conditions in the form of consensus sequences that define boundaries between introns and exons. Other examples include genetic recombination, transposons, translocation and other genetic variations. In addition, other rules that define gene regulation, DNA repair and alternative splicing are further examples of the complex language that makes up the genome. But this only emphasizes the formal nature of life's cybernetic prowess, and reinforces our point, that the linguistic-like effects could be defined by new production rules controlled by the proper grammatical syntax, thus expanding the genome language.

In terms of the genetic information contained within a gene, each codon selection is an occurrence of PI, since the sequential order of nucleotides, and then codons, is necessary for protein construction.

Information can be transferred from source to destination via an agreed-upon set of rules, and a language acted upon by algorithms. Each letter in the sentence "The glass contains water" is formally selected as a symbol from one of 26 alphabetical characters plus space. Each letter selection generates a simple form of Prescriptive Information (PI) as each letter contributes to forming a finite string of symbols, characterized as words having semantic meaning. PI is inherent in the selection of each letter from among 26 options even prior to the selection of words and word syntax. In both language and molecular biology synonyms occur where different letter selections can spell different words with the same semantic meaning. Sentence construction begins with letter selection. If a letter adds no significant meaning either to a word or to the contextual meaning of the sentence of which it is part, then that letter is not PI. Both letters in a word and nucleotides in the genome function as physical symbol vehicles (tokens) in a material symbol system (MSS) [23] and are forms of formal (non-physical) PI instantiation into physicality [24].

The question becomes, are the words "the," "glass," "contains," and "water" algorithms or data? Each word is composed of a linear sequence of symbols in the form of letters, which collectively transfer a greater meaning than the individual meaning of each character. This transfer is accomplished by defining semantic meaning to a prescribed sequence of letters the intent of which is to map meaning to an arbitrary sequence of tokens. This mapping is arbitrary as evidenced by the multitude of languages that exist in our world, each language mapping "meaning" to a multitude of arbitrary sequences of symbols or tokens, be it letters, shapes or pictures. This semiotic relationship transfers into biocybernetics and biosemiotics when viewed from the biological realm [36]. Since words are placeholders for an arbitrary mapping of "semantic meaning," they by themselves cannot perform or coherently instruct functionality without being combined in some structurally grammatical sentence. This deductively shows that words are not algorithms. Perhaps a demonstration of this is that one can find "words" in Scrabble pieces that are lined up randomly and turned over. But the strings are still random. The only thing that creates words out of these random strings is our minds algorithmically finding associations between letters based on language rules completely independent of the random sequences of Scrabble piece (token) letters.

Individual words have specific meanings. For instance the word "glass" means the material glass, but also has meaning in a general sense since it could refer to a drinking glass, window glass, etc. Some words can have multiple meanings depending on the sense in which they appear in context. For example the word "mean" could be interpreted as selfish or cruel or as another name for a mathematical average depending on its use in a sentence. As such, no individual words communicate information greater than itself. Since words can have ambiguous meanings, their meaning may be further dependent upon the structure of the sentence that they appear in. By structure, we mean the set of grammatical rules that define the construction of a sentence.

Sentences can be instructive since they contain two important properties. First they organize a thought instantiated through a collection of chosen letters and words to produce a product or function greater than the individual letters and words contained within them. Thus the sentence "The glass contains water" conveys the message of a glass vessel of arbitrary size containing an unknown amount of water. The more wisely chosen words we add, the more specific and possibly efficacious the message becomes. The purposeful addition of words to a sentence conveys more information as evidenced by the addition of detailed information contained in the sentence "The five ounce glass is filled with water." Secondly, by their own structure they have built in contingency that allows the outcome of their meaning to improve via the strategic placement of words.

### Genomic Information

In a gene, each nucleotide is a discrete 4 state configurable switch that can be represented using a material symbol system. The symbols A, G, T and C can be used to represent the string of quaternary (4-way) switch-settings found in a positive informational DNA single strand as discussed above. Highly conserved reference sequences represent discrete linear digital programming choices [12,13,23-25]. Each choice of symbol is made from an alphabet of four possible characters. This corresponds to a selection of each nucleoside from a space of four possible tokens. We emphasize again, that within a gene, nucleotides thus function as a physical symbol vehicle in a material symbol system [12,37,38]. From the perspective of the genome machinery, the nucleotides are a comma-less string of alphabetical characters. This is analogous to the discrete string of ones and zeros of magnetized regions on the computer hard drive.

From the perspective of protein synthesis machinery, the alphabet of nucleotides contained in the mature mRNA is read using the grammatical rule of organizing consecutive sets of 3 nucleotides in the form of triplet codons as illustrated above. A codon results in a 4-letter DNA alphabet translated to the twenty-letter alphabet in the protein space, letter for letter. Each codon is a Hamming block code consisting of three individual quaternary (four-way-knob) switch settings [24,39]. This Hamming redundancy code feature builds in noise-reduction properties allowing codons to become a more robust symbolic representation of each amino acid in protein space. Each codon is now defined as one of 61 arbitrary symbols mapped to the 20 amino acids constituting the protein space £. In addition there are three stop commands used for both the translation and transcription process. Within Ω_dna _is a finite space of codon block code symbols in the domain of the DNA language. These block code symbols are mapped to new letters in the protein alphabet (Ω_protein_) residing in protein language £. The reference frame (DNA/RNA or Protein) determines whether codons are considered to be words or letters. From the perspective of protein space, a codon is not a word, but a redundancy block code symbolizing each letter in the language of protein space. Each protein, like many words in the German language, is a very long word with many letters.

The confusion comes from translating an alphabet of 4 letters in the DNA/RNA language to an alphabet of 20 letters in the protein language. In this sense the triplet codons are not words, but schemas that incorporate Hamming redundancy block codes allowing protection against information loss in the Shannon channel. However, in the DNA world there are the equivalents of words composed from the 4 letter alphabet that have semantic meaning to the DNA machinery. For example, the three-letter codon TAA is equated to the word "stop". TAA, TGA and TAG are written in genome language (DNA language) and interpreted as stop or halt commands while UAA, UGA and UAG are equivalent commands in protein/RNA language as used in the protein/RNA machinery of the ribosome. Notice that you couldn't use the individual letters A, C, T, or G to represent individual functions like we do with the letters C and H on the faucet for Cold and Hot, without some similar icon that has a built in semantic meaning from which an interpretation could be surmised. PI is also inherent in one-letter selections like "H" and "C" on faucet handles, or "X" marks the spot on a map as they are context specific.

Abnormal translation termination is another example of a grammatical rule imposed upon the genome to minimize the loss of, or insertion of a base pair into an ORF (open reading frame) resulting in frame shift mutations (FSM). The immediate impact of a FSM is that they will code for incorrect amino acids costing the cell time, energy and raw material which could potentially produce toxic proteins. Interestingly, stop codons are distributed among the most common amino acids throughout the ORF due to a single frame shift. These overlapping sequences can be seen in the following example of a minus-one frame shift NNT | GAN (where N denotes any base pair and represents the comma-less break between codons). Notice that NNT and GAN could code Tyrosine and Glutamic acid respectively. The resultant minus-one frame shift produces the stop codon UAG (TAG) causing the ribosome to halt its protein construction. Imposing this grammatical rule that halt commands (in one of the three forms cited above) be inserted into every gene ensures that if a FSM occurs, the probable multiplicity of encrypted halt signs assists in preventing incorrect proteins from being synthesized. Less obvious is the fact that potential stop codons present during a FSM, are not seen in genes that exhibit no frame shift mutations. They are inconspicuous within the normal context of gene expression and yet become viable during erroneous FSM expressions without prematurely terminating non FSM codonic regions. This shows that the genetic code, in part, is constructed to lessen the impact of frame-shift errors due to the strategic use of grammatical rules. Thus the triplet configurations of codons in the ORF's buffer against, out of register protein synthesis due to frame shift errors.

There are many more examples of RNA words composed of many DNA/RNA letters that have meaning in the microRNA regulation process and identification of gene sections and organization.

### Language Mappings

Essentially, we propose the language of DNA domain (L) is mapped to the target space £ by:

$$\begin{array}{ccc} & L\overset{M}{\underset{}{\to }}\text{£}\;\text{where}\;M\;\text{is}\;\text{the}\;\text{mapping}\;\text{from}\;\text{DNA}\;\text{space}\;\text{to}\;\text{transcribed}\;\text{RNA}\;\text{space} & \\ & \left(\text{mRNA}\right)\;\text{to}\;\text{protein}\;\text{space}\;\text{via}\;\text{the}\;\text{maping}\;\text{in}\;\text{the}\;\text{form}\;\text{of}\;\text{tRNA}. \end{array}$$

However M can be decomposed into mapping β between DNA and pre-mRNA, mapping Đ between pre-mRNA and mature mRNA via alternative splicing and δ mapping mRNA into amino acids in the form of tRNA such that:

$$\begin{array}{ccc} & L\overset{M}{\underset{}{\to }}\text{£}=L\left(\text{DNA}\right)\overset{\beta }{\underset{}{\to }}\left(\text{pre}\right)\text{mRNA and}\;\left(\text{pre}\right)\text{mRNA}\overset{\text{Ð}}{\underset{}{\to }}\left(\text{mature}\right)\text{mRNA}\;and & \\ & \left(\text{mature}\right)\text{mRNA}\overset{\delta }{\underset{}{\to }}\text{£}\left(\text{protein}\right);where\;\delta \;is\;the\;mapping\;in\;the\;form\;of\;tRNA. \end{array}$$

It is interesting to note, a codon within a gene does not by itself produce the final protein. It does have individual formal meaning instantiated into its physical sequence. A gene or microRNA functions as a physical symbol vehicle syntax representing a string of choices [24]. As such, the linear digital sequence of codons is a form of PI [23,24]. Each codon transmits meaningful information which upon translation, can be equivocated to an arbitrary "letter" (in protein space) [12,40]. However, as a single letter it does not contain the equivalent meaning found in the language context of a word [24,39].

Once the rules or mappings are instantiated into physicality, then the physical codon sequence could potentially become a physical cause. Physicodynamic determinism is not the only kind of determinism. Choice-Contingent Causation and Control (CCCC) [19]) is also a valid form of determinism that can get instantiated into physicality. But, as we shall see, the process of translation is still not physicodynamically determined. Only formal algorithmic processing can bring about the process of translation within ribosomes.

Of course, grammatical rules used in protein synthesis are needed to interpret the nucleotide sequence and codon sequence within the genes of the DNA strand. Formal grammatical rules are a condition necessary for biofunction imposed on the genetic code. But note that this fact is not the result of any physicodynamic constraint. Obedience to these rules is not accomplished by cause-and-effect physicodynamic determinism. It is not "necessary" in the sense of physical law. It is necessary only in the sense that *if *the formal behavioral rules are disobeyed, formal functionality will be lost. Life chooses to obey the rules in order to survive. No physical law forces life to be alive.

It is only after edited mRNA is algorithmically processed in the ribosome--only after translation into polyamino acid sequence is complete, that cause-and-effect physicodynamic determinism becomes active or reigns. Only then does the sequence of R groups in amino acids largely determine thermodynamically the protein's folding into functional three-dimensional tertiary shapes. The rigidly-bound sequence of amino acids that was formally prescribed by CCCC determinism is the prime determinant of what the *average *minimum-Gibbs-free-energy sinks will be for that protein. How the globule forms in turn determines what bio-function will be produced. But this is only the final step (excluding for the moment the role of RNA and protein chaperones, which are themselves determined largely by CCCC). It is likely that even more formal controls of folding will be discovered. Models employing purely physicodynamic constraints have been very disappointing in predicting how proteins will fold. Most of the process of ribosomal protein manufacture is purely formal programming and algorithmic processing.

The language of the cell is posited to be formed by the set of words equivocated as proteins and RNAs. In the language of the ribosome, each codon is a symbol representing a letter of the amino acid alphabet ∑_protein_. The choice of symbolic representation is arbitrary as seen by various mappings of codon/amino acid groups such as in human mitochondria and other examples such as the codon UGA as tryptophan in the Mycoplasma species [41,42]. The successful summation of all the amino acids specified by a given mature mRNA form a word in protein space. Protein space is defined as the space of all functioning proteins [43]. The arrangement of such words is used to form bio-machinery, transduction circuitry and bio-signals used by the cell to communicate both internally and externally to its environment to sustain metabolic operations. The edited sequence of codons in mature mRNA largely prescribes not only the primary structure of the protein but also its secondary and tertiary (3D shape) structure[12], coupled with the assistance of other independently prescribed chaperone proteins. Each protein encoded in its associated gene is equivalent to a word of specific meaning [23,39] in protein space. Meaning is contained first in the prescribed amino acid sequence. The sequence of specific R groups determines the minimum-free-energy folding of the protein. Thus the prescribed sequencing blossoms into deeper layers of meaning. In molecular biological messages, "meaning" translates into successful "biofunction."

The functional sequencing of nucleotides in initial positive single nucleic acid strands is physicodynamically indeterminate (inert, decoupled from cause-and-effect determinism) [7,13,17,23,24,44-46]

All chemical bonds between nucleotides are identical 3'5'-phosphodiester bonds. Physicochemical factors cannot explain codon sequencing in single positive strands of DNA. Codon sequencing is formal, as is the editing of DNA transcriptions that produces mature mRNA. What does "formal" mean? Most of us would readily agree that language, mathematics, programming, and logic theory are all formalisms. But, do we understand why? The essential component of any formalism is the exercise of choice contingency, not chance contingency or necessity (law-like cause-and-effect determinism) [7,19,23]. Formalisms invariably employ purposeful choices and typically represent them using mathematical (e.g., 0 vs. 1 for binary decisions) or letter and word symbols for language. Each configurable switch-setting can be represented by a formal "on" vs. "off," "Yes" vs. "No," or "Open" vs. "Closed." Inanimate physicodynamics cannot make purposeful choices or participate in representationalism.

Symbol systems are governed by arbitrary rules, not laws. The rule could just as easily be that "1" represents "Closed" rather than "Open." Laws describe the invariant deterministic behavior of inanimate nature. Rules can be readily broken, and govern voluntary, choice-contingent behavior. All formalisms arise out of uncoerced choices in the pursuit of function and utility [7].

We propose that both the method used to combine several genes together to produce a molecular machine and the operational logic of the machine are examples of an algorithm which we will expand upon later. Molecular machines are a product of several polycodon instruction sets (genes) and may be operated upon algorithmically. But what process determines what algorithm to execute?

In addition to algorithm execution, there needs to be an assembly algorithm. Any manufacturing engineer knows that nothing (in production) is built without plans that precisely define orders of operations to properly and economically assemble components to build a machine or product. There must be by necessity, an order of operations to construct biological machines. This is because biological machines are neither chaotic nor random, but are functionally coherent assemblies of proteins/RNA elements. A set of operations that govern the construction of such assemblies may exist as an algorithm which we need to discover. It details real biological processes that are operated upon by a set of rules that define the construction of biological elements both in a temporal and physical assembly sequence manner. Small RNA's, peptides, short polypeptides, even other regulatory proteins can regulate genetic expression. Therefore codon syntax is only part of the PI that organizes and manages cellular metabolism. Sometimes non codonic nucleotide sequencing or even short polyamino acid sequencing (peptides sometimes have regulatory function) can be prescriptive.

In digital systems, algorithms are parts of software routines either embedded or called up in a program. In continuous systems, algorithms are analog in nature whose physical realization happens through the specific configuration of electrical circuits or mechanical assemblies. In the cell environment, we would propose that these algorithms are deductively called by a higher level of organization, possibly via software control or wet-wired as part of some type of automated control process. The rules that define an algorithm do not execute the algorithm. Something else does the operating according to a set of rules defined within the environment of which it operates. This is analogous to deciding what paragraphs in an instruction manual to read or when a specific algorithm is executed in the windows operating system. The action of when and what to read is accomplished by mechanisms outside the contents of the prescribed paragraphs/sentences or algorithm itself. Each sentence is composed of an arrangement of words, where each word is a physical symbol. But physical symbols are not algorithms. At best they may be a single instruction such as the word "stop". To get an algorithm, one would need to string together these symbols like what is done in the Chinese language. Just one "fit" contributes toward "instruction" and is PI, even though it's only one functional binary choice. Technically, one could have a program consisting of only one decision node purposeful choice. This would measure out to be one "fit" of FSC, or FI [25,26], assuming the bit marker provides opportunity for one functional binary programming decision to be recorded there.

### Algorithms

In order to determine if algorithms exist in biological systems, we need to define what an algorithm is. There are many definitions to describe algorithms. We choose to limit these definitions to those that most closely describe the algorithms used in computer science as defined in the background section above. We emphasize that this approach is justified by the analogous relationship that exists between a) computer functions, logic and code to b) linear discrete states and genetic code [10-12,14] that define biological systems seen in the DNA/RNA environment. An Algorithm is a set of rules or procedures that precisely defines a finite sequence of operations [27]. An algorithm starts with an input, initial state and produces an output [47]. Biological machines such as the ribosome input already algorithmically edited mRNA (PI) to operate upon, however an algorithm like a digital filter can have as its input, physical data, the nature of which may be some measured response from physicality (non PI). An algorithm can input either kind of data. These instructions prescribe a computation or action that, when executed, will proceed through a finite number of well-defined states either successively or recursively that leads to specific outcomes [47,48]. Most algorithms terminate at some final state but may also continuously loop producing outputs, as long as the system in which it resides is active. In this context an algorithm can be represented as: Algorithm = logic + control; where the logic component expresses rules, operations, axioms and coherent instructions. These instructions may be used in the computation and control, while decision-making components determines the way in which deduction is applied to the axioms[49] according to the rules as it applies to instructions.

**Figure 1:**
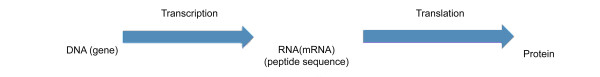
**Protein (peptide sequence)**.

In order to illustrate biological algorithms, we propose an algorithm representing the well-documented ribosome. A ribosome is a biological machine consisting of nearly 200 proteins (assembly factors) that assist in assembly operations, along with 4 RNA molecules and 78 ribosomal proteins that compose a mature ribosome [50]. This complex of proteins and RNAs collectively produce a new function that is greater than the individual functionality of proteins and RNAs that compose it. The DNA (source data), RNA (edited mRNA), large and small RNA components of ribosomal RNA, ribosomal protein, tRNA, aminoacyl-tRNA synthetase enzymes, and "manufactured" protein (ribosome output) are part of this one way, irreversible bridge contained in the central dogma of molecular biology [51] as shown in Figure 1 below.

The reason for the Central Dogma is ultimately *mathematical*, as Hubert Yockey points out [39]. The principle is not unique to molecular biology. The irreversible bridge of the Central Dogma is consistent with the one-way Configurable Switch (CS) Bridge that traverses the Cybernetic Cut [5,13,23] Formalisms' only access into physicality is to cross the CS Bridge from the far (formal) side of The Cybernetic Cut to the near (physical) side. Mathematically, there is no way to know from an amino acid alone which of the redundant codons prescribed that amino acid. There is always less information in a 20 character alphabet symbol system than in the 61 character alphabet symbol system from which it was 3:1 surjected with redundancy coding. Information is lost, the same as when we are only given the total sum from the roll of a pair of dice rather than the specific number on each die that was thrown. Information is always lost in codon to amino acid translation, never gained.

We propose that the ribosome be considered a builder of new three-dimensional meta-shapes through polymerizing each additional amino acid token shape, as opposed to adding numeric values. This summation of the monomeric sequence is not a one-dimensional object (sign and magnitude) as in computer space, but a sum projecting into real three-dimensional space (shape space).

This protein contains less linear digital PI than in the mRNA/gene polycodon. The reason is that the polyamino acid string does not tell us which of the redundant codons prescribed each amino acid. That information is lost in the process. Some might argue that the Gibbs free energy sinks that come into play after the R group sequence of the polyamino acid sequence (the protein's primary structure) is established creates new dimensions of PI not prescribed by the linear digital prescription of the mature mRNA.

This perspective is hard to defend, however, since thermodynamics and an inanimate environment cannot make programming choices at decision nodes required to generate new PI. Choice-Contingent Causation and Control (CCCC) is essential to generate new PI [19].

One of the greatest enigmas of molecular biology is how codonic linear digital programming is not only able to anticipate what the Gibbs free energy folding will be, but it actually prescribes that eventual folding through its sequencing of amino acids. Much the same as a human engineer, the nonphysical, formal PI instantiated into linear digital codon prescription makes use of physical realities like thermodynamics to produce the needed globular molecular machines.

We hypothesize that the functional operation of the ribosome consists of logical structures and control that obeys the rules for an algorithm. The simplest element of logical structure in an algorithm is a linear sequence. A linear sequence consists of one instruction or datum, followed immediately by another as is evident in the linear arrangement of codons that make up the genes of the DNA. Branching control or routines are another form of logical structure. Branching allows control of the execution routine to jump to a different part of the algorithm. Other logical structures such as conditional control direct the execution of the algorithm's flow based on a set of variables meeting some condition or rule. This means that the linear sequential execution of the algorithm is broken and the execution path is branched to some other instruction or continues in its original path dependent on how the condition is evaluated. The actions of the spliceosome can be thought of branching away from the linear sequence of codons when it detects introns which are then cut out.

In computer systems, transistor circuits are configured to form logical gates. The arrangement of transistors and their resulting functions, prescribe an instantiation of PI into physicality [7,10-12,46,52,53]. Both logic and functionality are non physical formalisms. Both can be instantiated into physicality using logic (electrical/optical) gate settings. Instantiation means the programming of non physical formal choices into physicality. This is usually accomplished through the formal setting of physical configurable switches, the selection of physical symbol vehicles (tokens) from an alphabet of physical tokens (e.g., Scrabble pieces), or through the choice-contingent integration and organization of component physical parts into holistic devices and machines [17]. DNA bases are physical tokens. They can be formally arranged into functional linear digital sequences of Dbit (dual bit based quaternary decision node) instructions. The resulting syntax of tokens is a form of instantiation of formal prescription into physicality provided that algorithmic processing is also prescribed. Upon algorithmic processing, logical organic circuits are arranged and assembled using genetic engineering.

**Figure 2:**
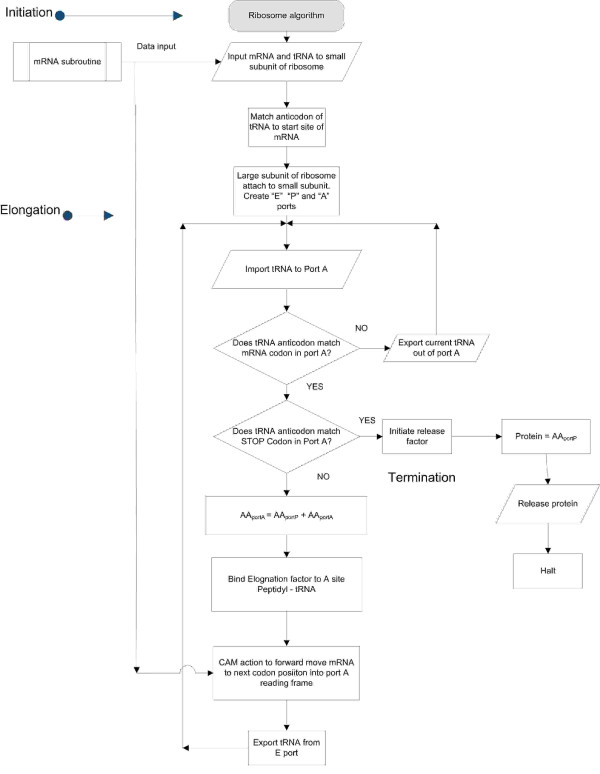
**Proposed Ribosome Algorithm**.

The set of rules governing programming choices must obey the three classic laws of logical thought shown below if formal function is to be expected. As in the case of computer circuits, this does not preclude physical law determinism within the electrical switch environment. But physical law determinism alone has never been observed to generate non trivial formal pragmatism. Expedient thought obeys the "law" (technically a rule) of non contradiction, i.e., it is not possible for something to be true and not true at the same time and in the same sense. The statements that describe the functionality of the ribosome in the proposed algorithm of Figure 2 obey this rule of logical inference as well as the identity rule prohibiting Excluded Middle [54].

### Ribosome Algorithm

Biological systems and their development have made use of the theory of algorithms and computability (Automata and Biology). Part of algorithmic theory deals with general principles of operation and structure of automata [55]. We have chosen to analyze the behavior of the ribosome mechanism without regard to ways in which this mechanism is realized. The basic relations that govern the ways in which ribosomes' operate can be described with rules and logic. The basic ways in which finite state automata process information can be exhibited in certain biological machines such as the ribosome in order to extract behaviors and computability [29].

The primary function of the ribosome can be described at the top level as three main functions defined as Initiation, Elongation and Termination [56]. Examination of the ribosome functionality is captured and modeled in the proposed algorithmic function (at a minimum) shown in the flow chart of Figure 2. The ribosome algorithm (R-algorithm) is composed of a logical sequence of commands and decision-nodes choices. These programming decisions mimic a discrete program, acting upon inputs such as tRNAs, aminoacyl-tRNA synthetase enzymes and mRNA while producing outputs including empty tRNAs (de-acylated tRNA) and proteins. Each block shown in the flow chart of figure 2 may be instantiated into the product structures of proteins and RNA's selectively and cybernetically. The blocks are sequence-dependent to achieve logical functions. What is not shown is the need for different, independent aminoacyl-tRNA synthetase enzymes which must all be there for the ribosome to produce any protein. Close examination of the algorithm in figure 2 shows that the mRNA (which is itself a product of the gene copy and editor subroutine) [57] is a necessary input which is formatted by grammatical rules as discussed previously enabling the proposed ribosome algorithm to successfully execute, producing a requested protein. The mRNA attaches to the small subunit of the ribosome followed by both the recognition and attachment of the initiator tRNA to the start codon (usually AUG) of the mRNA located in the "P" site of the ribosome. This is followed by the docking of the large subunit of the ribosome, along with participating initiation factors to form the complete ribosome [15]. These steps are captured in the initiation section of the R-algorithm. The ribosome contains three ports designated "A", "P" and "E". The input tRNA is contained in the "A" port or data site, where it is checked to see if its anticodon tRNA matches the mRNA data codon [58]. The P site contains the previous tRNA along with the growing amino acid chain, while the E port contains de-acylated tRNA that preceded the tRNA in P port. The action of comparing the anticodon tRNA with the current selected mRNA codon in the "A" port reading frame represents a decision node. It's what *precedes *this final bonding that requires the instantiation of a great deal of PI within the system--the physicodynamically inert (indeterminate) sequencing of codons, the linking up of the correct amino acid with the correct tRNA for the codon-anticodon system to work, the specificity of each amino-acyl tRNA synthetase, etc. A contrived system must exist to instantiate the decision and action process detailed in the R-algorithm whose comparative result redirects the algorithm flow to either be an iterative action or continue to the next successive step. This is a cybernetic process. The next successive step is also a decision node whose comparative result either halts the program or continues to the next successive step based on the detection of the stop codon, again, another cybernetic decision affecting the execution vector of the program flow. Upon successful matching of codon to anticodon in the "A" port, the amino acid chain from the nascent tRNA in the "P" port is added to the amino acid in the "A" port which is modeled as:

(3) $$\text{A}{\text{A}}_{\text{portA}}=\text{A}{\text{A}}_{\text{portP}}+\text{A}{\text{A}}_{\text{portA}}$$

where AA_portA _is the current amino acid in Port A

AA_portP _is the amino acid chain in port P including the initial condition normally defined as amino acid methionine from the initiator tRNA representing the start codon.

The completion of amino acid addition is followed by what we perceive as cybernetic action of advancing the mRNA input via three discrete steps by mechanically ratcheting the ribosome along the mRNA track [59]. Details of this mechanical action are a little fuzzy but can be modeled with descriptive logical statements as shown in Figure 2. The current states of the machine shows that empty tRNA in port E is expelled out of the machine, the contents of port P are translocated into Port E and amino acid chain of current tRNA in port A is translocated into port P. Notice that the R-algorithm will output any sequence combination or any syntax of amino acids as encoded in the mRNA present at its input port, regardless of whether the encoded amino acids produce a functional or non functional protein. In our view the ribosome is a machine that executes a sequence of discrete instructions operating upon a set of arbitrary discrete codon packages (PI data) producing a protein product as its output. The machine can produce any variation of protein product by simply changing the syntax of both the tRNA (anti-codon/amino acid map) and the DNA codons. This property allows the R-Algorithm to universally produce any linear amino acid sequence product. The machine also makes calls to local memory defined as tRNAs. The tRNAs are necessary to implement the logical structure of the "conditional control" decision node. The temporary storage of the forming amino acid chain is contained in port P. Port P contains all states of the protein synthesis process.

This initial simplistic comparison does not preclude later comparisons with artificial automata on many additional levels, layers and dimensions, including reading in both directions, regulatory microRNAs arising from the complementary strand to then regulate the coding strand, etc.

The tRNAs are necessary to implement the logical structure of the "conditional control" decision node. The temporary storage of the forming amino acid chain is contained in port P. Port P contains all states of the protein synthesis process. The tRNA is more than memory, as it selects the matching amino acid. Perhaps its RNA is a prescriptive selection program that is processed by associated proteins that form a separate computer system capable of interacting with the ribosome system--the tRNA output becomes another ribosome input. The above features define the ribosome complex in a greatly reduced and naïve form as a Turing machine. A Turing machine, however, should be able to simulate the logic of any computer algorithm. If the ribosome can only execute the logic of one particular algorithm, can we still call it a Turing machine? "Turing complete" (TC) doesn't require that all computables are implemented, but that they could be given the hardware/software of the system. Boneh et al. [60] proved that DNA-based computers are TC in 1996. But then the next question would be, "Does the ribosome have the necessary hardware/software to potentially implement *all *computables?"

Babbage's Analytical Engine was proved to be Turing Complete, as was a theoretical machine having a single instruction. Since the conditional controls are implemented using tRNA, the Turing completeness may depend on structures external to the ribosome itself. The components are manipulatable [61].

Recently, researchers have "boosted the number of amino acids that can be built into a "protein" from the 20 covered by the existing genetic code to 276. That's because Chin's new code [62] creates 256 possible four-letter nucleotide words or 'codons,' each of which can be assigned to an amino acid that doesn't currently exist in living cells... Chin's team redesigned several pieces of the cell's protein-building machinery, including ribosomes and transfer RNAs (tRNAs). Together, they read the genetic code and match it up to amino acids" [63,64].

Theoretically, since the mRNA, as well as the components of the ribosome along with all of life's other components, are manufactured via proteins, which are constructed by the ribosome, it would be possible to encode proteins necessary to perform math or logical functions (such as done in Boneh's DNA computers), so that the ribosome system could possibly be viewed as TC. Certainly, life has no need to do math for the sake of computation (which is one aspect of TC). Evidence does exist that life's machinery might be able to do such computations [65]. But since the subject of Turing completeness of ribosomal systems is not the focus of this paper, we shall simply point out that the flexibility of ribosomal systems is seen to be much greater than originally suspected.

## Discussion

The question becomes, "Does the mRNA instruct the ribosome, or is it just a prescriptive informational data feed?" Notice that *the algorithm of the ribosome *is not altered in any way in producing a product as defined by the prescriptive data stream of the mRNA. *From the perspective of the ribosome*, it is simply waiting for data to execute its program. Its programming does not change and all it sees is input data and all it produces is output data. This data is acted upon according to the PI contained in the ribosome's own logical structure. The ribosome executes decisions as illustrated in the two decision blocks of Figure 2, suggesting that instructions are contained in the sequencing and configurations of the many proteins and RNA in the ribosome itself, independent of the PI data feed.

The logical mappings (codon to amino acid) that are performed are undeniably cybernetic. The sequence of instructions *in the ribosomal proteins and RNA *meets the criteria of an algorithm given in the introduction and proceeding section. The PI data feed gives no instructions to the ribosomal operation, only to the protein product. For example, the PI data feed gives no command to the ribosome to polymerize an amino acid to the product chain. The instruction to "add" a monomer to the polyamino acid output is inherent in the independent ribosomal algorithms. But the question of "Which particular amino acid?" to add can only be answered by investigating a synergism of PI's from multiple sources:

1) the data stream

2) the tRNAs that link anticodon on one end to the "correct" amino acid on the other end

3) the sequence and conformation of each aminoacyl-tRNA synthetase

4) the algorithmic processing by the ribosome

The R-algorithm satisfies the rule, Algorithm = data + Control, generating machine states as shown below. These statements proposed are therefore logical statements. Their decision capability thereby grants full control of the system. This proposed formal organization enables the functions of the R-algorithm to be hardware implemented.

Machine states of the ribosome are as follows, where n = the machine step relative to translocation action:

1. tRNA (n-2) in Port E to be expelled

2. tRNA (n-1) in Port P contains previous amino acid chain

3. tRNA (n) in Port A is current amino acid

By comparison, the electrical circuit configuration of logic gates in a microprocessor functions in the same way. The data feed does not contain instructions with electrical circuits nor does mRNA in cellular cybernetics (with the possible exception of the stop codons).

Formal rules govern the hardware functionality of computers through the hardware instantiation of logical algorithms. In computers, firmware accomplishes boot-up procedures that allow the operating system to communicate with input/output devices. This set of software that is executed upon boot-up loads the operating system and translates operating system calls into the language of input/output devices such as keyboards disk drives and monitors. It has been proposed that a similar set of formal rules may be instantiated into cellular wetware circuitry (mentioned below) to model the operational behavior of the cell [57]. A comparative analysis between a computers central memory and that of eukaryote DNA strongly suggests that DNA and its molecular machinery operate as a central biological memory system serving as a repository of cellular information. In this analogy the DNA functions at a minimum as a biological hard drive operating within its nuclear environment. It has been proposed that the cellular circuits used to request prescribed data use a biological equivalent of firmware as used in computers systems [57]. This firmware may exist in the instantiation of transduction pathways (possibly in the cytosol) that transfers cellular requests for proteins into the RNA language of the nucleus/DNA. Also, epigenetic processes would seem to behave like firmware. In this analogy, epigenetic processes boot up the cell. This would enable the histone code leading to stem cell differentiation [57].

Wetware in cells is equivalent to the logical gates, communication circuits and other gate structures that define microprocessor hardware. On the biological side the analogy would include the transcription factors, transduction molecules and other combinations of protein and RNA molecules in which their combined patterns perform functions. Comparing computer science with life doesn't mean that we have to maintain an analogy at every point. Differences in data format and instructions whether implicit or explicit (as in stop codons) may be giving us clues as to the functionality of biological operating systems in living systems. How all this came to be is a subject of intense research in such fields as ProtoBioCybernetics and ProtoBioSemiotics [7]. ProtoBioCybernetics is the study of how initial bona fide controls (not just mere constraints) emerged in the first primitive protocells to steer physicochemical events toward formal organization and eventual biofunctionality. ProtoBioSemiotics studies how initial communication systems developed within and possibly between protocells.

### mRNA Characterization

By contrast, the mRNA neither makes decisions nor alters any programming direction path (execution vector) within itself or alters the program structure, computation or control of the R-algorithm. This is because there is no contingency to interpret mappings and no path to intrinsically implement control changes to the ribosome within the mRNA formal structure. It could be argued that the mature mRNA contains recorded programming choices "already made." It is likely that mRNA is the result of some other program involving a series of pre-recorded programming choices. The point at hand, however, is that the mRNA by itself does not command or make programmable decisions. All of that capability resides in the ribosome. In other words, mRNA is not executable by itself. These properties, or lack thereof, give the mRNA the characteristic of prescriptive programming data, equivalent to the machine code data stored in a computer's memory. Since the codons and their constituents represent choices that are neither a product of physical law nor chance contingency [23], they represent specific choices of PI manifested as configurable switch settings. We must make careful distinction between "configurable switches" and "configurable switch-settings." "Configurable switches" are purely physical, whereas "configurable switch settings" are purely formal. This is what ultimately defines The Cybernetic Cut [23]. Configurable switch settings are symbolic representations of protein prescription (specifically selected physical symbol vehicles; tokens), and therefore are an instance of prescribed data (non-physical, formal PI). One could argue that the promoter sections and regulatory components of the gene are part of a gene algorithm. However, all of these functions may be considered as a combination of meta data (promoter sequences and histone code identifying gene status), location tags and other formatting structures present in every gene. The format identifies the location, alignment and initiation start site location for the RNA polymerase II read head along with other regulating functions of an equivocated chromosome disk drive known as the DNA Hard Drive [57].

### DNA characterization

We make the argument that the genome operates using a language based system composed of alphabetical strings of nucleotides forming words and constrained by grammatical rules. We have shown that codons are strings of alphabetical nucleotides that are encoded to allow the 20 character (amino acid) alphabet of the protein language to be represented using the 4 character alphabet of the DNA language. However, we posit that the genome is composed of words in the form of regulatory RNA's, linRNA's etc. along with consensus sequences such as the TATA box, promoter, enhancer and insulator sequences that are recognized by the genome machinery. We posit that there are additional rules of grammar, other than the triplet rule for codons that defines the rule allowing overlaid and multilayered genes, reverse transcription, alternative splicing and epigenetic operations.

A gene may be considered to be part of a subroutine [25] within a larger complex cellular software algorithm. Each gene contains both words such as its promoter regions and data in the form of codons. The subroutine is acted upon when executed within the DNA environment. We posit that the DNA language exists based upon coherent orderly transcription, editing, error detection, repair and genome duplication processes involving recognition of reading genome sequences. These consensus sequences exhibit semantic functionality as defined by the interactions between sequences and the bio-machinery in the nucleus. The alphabet, words grammar and language models we developed fit within the Automata models developed earlier.

## Conclusion

The concept of Prescriptive Information has been examined as related to the ribosome, mRNA and in part to the genome. We have shown that there is a dichotomy within the definition of Prescriptive Information resulting in a differentiation between prescribed algorithms and prescribed data. Examination of mature mRNA in eukaryotic cells reveals no executable path or mechanism for control within its contents. Since there is no mechanism for control, there is no contingency for mRNA to execute any kind of algorithmic process (other than a simple sequence), and therefore mRNA is defined as prescriptive data satisfying the data component of the purposed dichotomy definition. This definition covers all mRNA sequences and has extensions to genes themselves with respect to the codons defined in their reading frame. Furthermore, by viewing the amino-acid sequence in protein space, we have shown that it is representative of letters which combine to form extremely long words (proteins). Since words have been shown not to be algorithmic, this enforces our claim that both the information in each protein word (language £ ) and its mapping back into sequential DNA Hamming block codes (alphabetical strings of language L) are instantiations of prescribed data. For these reasons, a gene (reading frame section) and its associated mRNA is not an algorithm. But yet, it is an instance of Prescribed Information because it contains a prescription of specific order of the base 4 digital language representing a protein or RNA product. We would add the caveat that the start and stop codons can be considered as boundary conditions defining the beginning and the ends of the data set thus representing an implicit command recognized by the ribosome. The polycodon symbolically prescribes the amino-acid sequence of each protein primary structure while the transcription procedure is a bona fide formal decryption governed by rules, not invariant physical laws.

An operational analysis of the ribosome has revealed that this molecular machine with all of its parts follows an order of operations to produce a protein product. This order of operations has been detailed in a step-by-step process that has been observed to be self-executable. The ribosome operation has been proposed to be algorithmic (R-algorithm) because it has been shown to contain a step-by-step process flow allowing for decision control, iterative branching and halting capability. The R-algorithm contains logical structures of linear sequencing, branch and conditional control. All of these features at a minimum meet the definition of an algorithm and when combined with the data from the mRNA, satisfy the rule that Algorithm = data + control. Remembering that mere constraints cannot serve as bona fide formal controls, we therefore conclude that the ribosome is a physical instantiation of an algorithm.

There is a synergy between the machinery of the ribosome and its coherence with the language context of the DNA/RNA environment, reinforcing the prescribed algorithmic operations of the ribosome. There is no known physicodynamic cause for the codon to tRNA translation scheme. Since all genes can be modeled using rules (be they grammar or logical) rather than physicodynamic determinism, we inductively assert that the operation and organization of the genome operate under the influence of a programming language. The genome can be considered as a collective ensemble of instructions and data. Portions of the DNA sequences are algorithmic instantiations. This is evidenced for example, by pre-initiation, enhancer and promoter regions, lincRNA's, siRNA's and a host of other instructive sequences, that collectively instruct direct functionality such as gene regulation. In addition to the instruction constructs, the genome is also composed of data in the form of codons. This results in mature mRNAs that are handled as data by other processors (ribosome) which are executing their own algorithms. In other words there are "multiple programming languages" in the cell.

RNA Polymerase II performs many tasks in order to copy genetic information. Included are generating gene copies and regulatory RNA structures. There are at least 12 subunits in human RNA polymerase that must assemble together in the proper order and in readable locations (promoter sites) upon the genome. As such, there exist multiple transcription factors that work coherently within the DNA system to locate and set up the pre-initiation sites from which the RNA polymerase will assemble. Once assembled, RNA polymerase waits for a start signal at which time it begins to unwind the double helix strand and reads the genetic data from which a copy is made during the elongation process. At the end point of the gene, the copy process is terminated. As the RNA polymerase advances along the DNA strand, it must select the appropriate RNA nucleotide that is paired with the current complementary DNA base. Successful selection may be part of a decision process leading to the next sequential nucleotide in the DNA sequence. The way in which RNA polymerase initiates the transcription process to locate, copy complementary data and terminate the copy process strongly suggests a formal procedure of operations that is algorithmic. Although the determination of whether or not RNA polymerase is algorithmic is beyond the scope of this manuscript, it lends itself to modeling as an instantiation of still another CCCC prescribed algorithm.

The correlation between linguistic properties examined and implemented using Automata theory give us a formalistic tool to study the language and grammar of biological systems in a similar manner to how we study computational cybernetic systems. These examples define a dichotomy in the definition of Prescriptive Information. We therefore suggest that the term Prescriptive Information (PI) be subdivided into two categories: 1) Prescriptive data and 2) Prescribed (executing) algorithm.

It is interesting to note that the CPU of an electronic computer is an instance of a prescriptive algorithm instantiated into an electronic circuit, whereas the software under execution is read and processed by the CPU to prescribe the program's desired output. Both hardware and software are prescriptive.

## Abbreviations used in this paper

(PI): Prescriptive Information; (MSS): Material Symbol System; (CCCC): Choice-Contingent Causation and Control; (fit): functional bit; (FSC): Functional Sequence Complexity; (FI): Functional Information; (DI): Descriptive Information; (R-Algorithm): Ribosome algorithm; (FSM): Frame Shift Mutation.

## Conflicting interests

The authors declare that they have no competing interests.

## Authors' contributions

DJD conceived the overall concept including generation of proposed ribosome algorithm. DJD managed iterative refinement of this review from input provided by co-authors. DLA and DJ provided major insight into subject matter contributing to the technical content and refinement of the manuscript. All authors contributed to writing the manuscript.
